# The association between hospital frailty risk score and adverse inpatient outcomes in older adults with colorectal cancer

**DOI:** 10.1038/s41416-026-03385-2

**Published:** 2026-05-06

**Authors:** Hao-Tsai Cheng, Chen-June Seak, Ching-Yi Cheng, Shu-Wei Huang, Chang-Mu Sung, Tsung-Hsing Chen

**Affiliations:** 1 Division of Gastroenterology and Hepatology, Department of Internal Medicine, New Taipei Municipal TuCheng Hospital (Built and Operated by Chang Gung Medical Foundation), New Taipei City, Taiwan, ROC; 2 Division of Gastroenterology and Hepatology, Linkou Chang Gung Memorial Hospital, Taoyuan, Taiwan, ROC; 3 Chang Gung University College of Medicine, Taoyuan, Taiwan, ROC; 4 Graduate Institute of Clinical Medicine, College of Medicine, Chang Gung University, Taoyuan, Taiwan, ROC; 5 Department of Emergency Medicine, New Taipei Municipal TuCheng Hospital (Built and Operated by Chang Gung Medical Foundation), New Taipei City, Taiwan, ROC; 6 Department of Emergency Medicine, Linkou Medical Center, Chang Gung Memorial Hospital, Taoyuan, Taiwan, ROC; 7 Graduate Institute of Health Industry Technology, Research Center for Chinese Herbal Medicine and Research Center for Food and Cosmetic Safety, Chang Gung University of Science and Technology, Taoyuan, Taiwan, ROC; 8 Department of Pulmonary Infection and Immunology, Chang Gung Memorial Hospital at Linkou, Taoyuan, Taiwan, ROC

**Keywords:** Cancer therapy, Cancer

## Abstract

**Background:**

Impact of frailty on prognosis in patients with metastatic and non-metastatic colorectal cancer (CRC) was studied.

**Methods:**

Patients aged ≥60 years with CRC were identified in Nationwide Inpatient Sample database and analyzed retrospectively. Frailty was defined when Hospital Frailty Risk Score ≥5. Patients were grouped and matched by metastatic status. Logistic and linear regression were used to assess association between frailty and in-hospital outcomes.

**Results:**

After matching, 99,017 metastatic and 418,435 non-metastatic CRC were included. Frailty was significantly associated with increased in-hospital mortality (metastatic: OR = 1.10, 95% CI 1.05–1.17; non-metastatic: aOR = 1.05, 95% CI 1.00–1.10), prolonged length of stay (metastatic: OR = 1.30, 95% CI 1.26–1.34; non-metastatic: aOR = 1.37, 95% CI 1.34–1.39), and discharge to long-term care (metastatic: OR = 1.67, 95% CI 1.62–1.73; non-metastatic: aOR = 2.10, 95% CI 2.07–2.14). Frailty was also associated with higher total hospital costs, with additional $3,750 (95% CI $2940–$4560) in metastatic CRC and $1920 (95% CI $1480–$2360) in non-metastatic CRC.

**Conclusions:**

Frailty is an independent predictor of adverse outcomes among older patients with CRC, regardless of metastatic status.

## Introduction

Colorectal cancer (CRC) is the third most common cancer worldwide. Curative treatment consists of surgical resection, combined in many cases with chemotherapy and/or radiation [1, 2]. The incidence of CRC increases with age, with a median age at diagnosis of 72 to 75 years. In 2020, nearly 900,000 people died from CRC annually [3]. Despite older age or having multiple comorbidities, most older patients undergo surgery for CRC. Expectedly, older patients are at higher risk than younger patients for postoperative complications and mortality, especially within the first year after surgery [4]. In addition to the demographic features, in developed countries, lifestyle factors such as obesity, low physical activity, and smoking also increase the risk of CRC [5].

Frailty is defined as increased vulnerability to stressors and declines in multiple physiological systems, resulting in an increased risk of negative health outcomes such as falls, hospitalisation, and mortality [6, 7]. While commonly associated with aging, frailty more often occurs in older adults with chronic illnesses [8]. Various tools have been developed to identify frailty, including the well-known Fried phenotype, which classifies individuals as robust, prefrail, or frail based on clinical criteria such as weight loss, fatigue, and slow gait [9]. An earlier study reported that approximately 10% of adults over age 65 and up to 50% of those over 85 are considered frail [10].

The Hospital Frailty Risk Score (HFRS) was developed to identify frailty using International Classification of Diseases (ICD)-coded hospital records, making it particularly practical for claims-based retrospective studies [11]. Compared with the Fried phenotype and other bedside assessment tools, the HFRS enables efficient identification of frailty using administrative data and has been validated across multiple inpatient populations [12–14].

In CRC, frailty assessment is especially important, as older adults comprise the majority of patients, and frailty may independently affect treatment decisions and clinical outcomes. Evidence has shown that frailty is associated with worse postoperative outcomes in CRC patients, including longer hospitalisations, increased complications, and higher mortality [7, 15]. However, most existing studies have focused on surgical cohorts and have rarely assessed the impact of frailty using the HFRS in a nationwide inpatient population. Additionally, few have differentiated between metastatic and non-metastatic CRC. Therefore, the aim of this study was to provide a comprehensive, nationwide picture of how frailty, as measured by the HFRS, influences short-term outcomes and healthcare utilisation—including in-hospital mortality, length of stay (LOS), discharge to long-term care, and total hospital costs—among older adults hospitalised for CRC. The findings from this study may help guide risk stratification and clinical decision-making, and offer insights to optimise inpatient resource allocation and care planning for frail CRC patients.

## Methods

### Data source

This population-based, retrospective observational study extracted all patient data from the Nationwide Inpatient Sample (NIS) database, which is the largest all-payer, continuous inpatient care database in the United States (US), having data of about 8 million hospital stays each year [16]. The database is administered by the Healthcare Cost and Utilisation Project (HCUP) of the Agency for Healthcare Research and Quality (AHRQ) (https://www.hcup-us.ahrq.gov/db/nation/nis/NIS_Introduction_2020.jsp). This continuous, annually updated database derives patient data from about 1,050 hospitals in 44 states in the US, representing a 20% stratified sample of US community hospitals as defined by the American Hospital Association. The data contain primary and secondary diagnoses, primary and secondary procedures, admission and discharge status, patient demographics, expected payment source, duration of hospital stay, and hospital-related characteristics (i.e., bed size/location/teaching status/hospital region).

### Study population

Data of hospitalised older adults aged 60 years or older, who were admitted with CRC diagnosis between 2005 and 2018, were extracted from the NIS database. CRC diagnoses were confirmed based on the International Classification of Diseases, Ninth and Tenth Revisions, Clinical Modification (ICD-9-CM and ICD-10-CM) codes: 153, 154.0, 154.1, V10.05, V10.06, C18-20, Z85.03, and Z85.04. Hospitalisations were eligible if the CRC codes appeared in the principal or any secondary diagnosis field. Patients with missing information on key outcomes and variables (i.e., mortality status, length of stay [LOS], discharge destination, total hospital costs, sex, household income, primary payer, admission type, hospital bed size, and race/ethnicity), as well as those missing sample weight values, were excluded. The patients were further grouped into non-metastatic and metastatic CRC (ICD-9-CM: 197-198 or ICD-10-CM: C78-79), and then divided by whether or not they had frailty.

### Study variables

#### Study outcomes

Primary study outcomes were: (1) in-hospital mortality; (2) prolonged LOS, defined as a LOS >75th percentile; (3) discharge to long-term care facilities, defined as discharged to a nursing home or long-term facility; and (4) total hospital costs.

#### Assessment of frailty

Frailty was assessed using the HFRS, a validated claims-based frailty measure derived from a predefined set of ICD diagnostic codes that serve as surrogates for frailty-related conditions, such as volume depletion, chronic pulmonary disease, and heart failure [15]. The HFRS has been widely validated and applied across diverse clinical settings and health systems internationally [17]. Because the NIS is a hospitalisation-level database and does not allow longitudinal linkage across admissions, frailty-related ICD diagnostic codes were identified exclusively from diagnoses recorded during the index hospitalisation. Hospitalisations occurring before October 1, 2015 were coded using ICD-9-CM, whereas those on or after this date used ICD-10-CM, in accordance with the nationwide transition in coding systems.

The HFRS was calculated by applying the original weighting scheme, in which each frailty-related ICD diagnosis code is assigned a predefined weight reflecting its relative contribution to frailty, and the total HFRS is obtained by summing the weights of all eligible diagnosis codes recorded during the index admission. Consistent with prior studies, patients with an HFRS ≥ 5 were classified as frail, whereas those with an HFRS < 5 were classified as non-frail [18]. The HFRS was calculated by applying the original weighting scheme to diagnosis codes recorded during the index admission only. To ensure methodological transparency and reproducibility, the complete lists of ICD-9-CM and ICD-10-CM diagnostic codes and their corresponding weights used to derive the HFRS are provided in Supplementary Tables 5 and 6.

#### Covariates

Data from patients’ demographic characteristics, including age, race/ethnicity, household income, insurance status (primary payer), admission types (elective or emergent), and severity of illness were extracted from the NIS database. Patient’s clinical characteristics, including obesity, active tobacco use, major comorbidities (ischaemic heart disease, congestive heart failure, diabetes, cerebrovascular disease, chronic pulmonary disease, severe liver disease, moderate-to-severe renal disease, and systemic connective tissue disorders), and severity of comorbidities assessed by Charlson Comorbidity Index (CCI), was also identified through ICD-9 and ICD-10 codes [19]. Detailed ICD codes used are provided in Supplementary Table 1. Hospital-related characteristics (bed size, location/teaching status, and hospital region) were extracted from the database as part of the comprehensive data available for all participants in accordance with other studies in the literature that have used the NIS data.

To account for treatment-related differences, CRC-directed surgical procedures were identified using ICD-9-PCS and ICD-10-PCS codes and classified as: (1) open surgery, (2) laparoscopic/minimally invasive surgery, (3) liver resection for metastatic disease, and (4) no CRC-related surgery (i.e., no colorectal or liver metastasectomy performed during the admission). Additionally, the five most common principal diagnosis codes of the hospitalisations have been reviewed, as presented in Supplementary Table 7.

### Statistical analysis

The NIS database includes a 20% sample of US annual inpatient admissions, weighted samples (before/after 2012 using TRENDWT and DISCWT), stratum (NIS_STRATUM), and cluster (HOSPID) were used to produce national estimates for all analyses. The SURVEY procedure in SAS performs analysis for sample survey data. Descriptive statistics are presented as numbers (n) and weighted percentages (%), or as means and standard errors (SE). Group comparisons use standardised mean difference (SMD). The propensity-score matching (PSM) method uses the SAS OneToManyMTCH macro to balance the baseline characteristics of patients with and without frailty. The macro prioritises “best” matches first and then proceeds with “next-best” matches until no more can be made. The patient cohort was matched at a ratio of case (frail): control (non-frail) = 1:1 based on variables with SMD ≥ 0.1 in Supplementary Table 2 (except for HFRS).

Univariate and multivariable logistic regression analyses were used to determine associations between the study variables and binary outcomes, while linear regression was used to assess associations with the continuous outcome (i.e., total hospital costs). In addition, stratified analyses on the impact of frailty on in-hospital mortality were performed according to age, sex, and race/ethnicity. An SMD < 0.1 indicates a balanced distribution of variables between groups. Variables with an SMD ≥ 0.1 after PSM were additionally adjusted for in the multivariable model. All p-values were two-sided, and *p* < 0.05 was considered to represent statistical significance. All statistical analyses were performed using the statistical software package SAS software version 9.4 (SAS Institute Inc., Cary, NC, USA).

## Results

### Patient selection

**Fig. 1:**
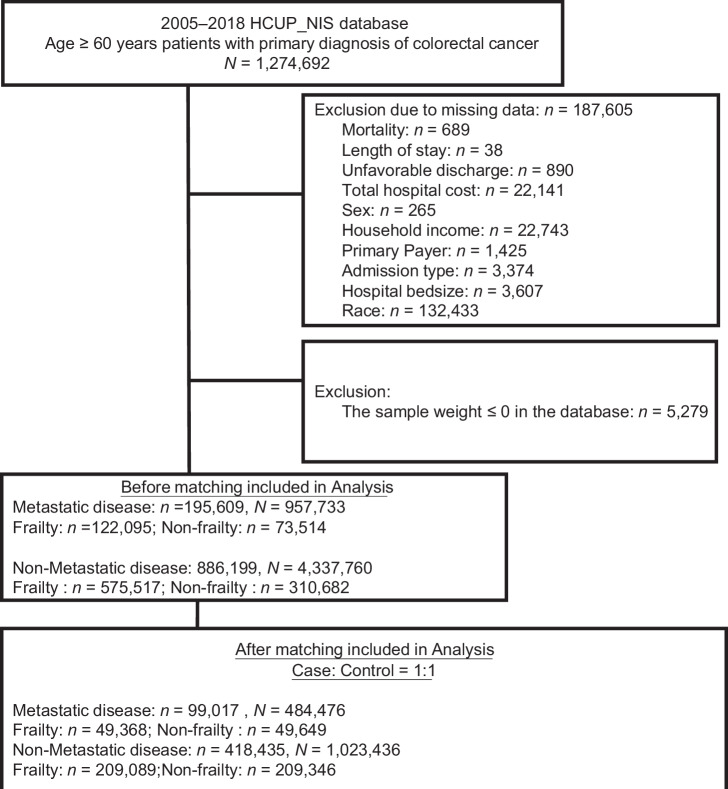
Flow diagram of patient selection. A total of 1,274,692 patients were initially identified from the NIS database. After excluding 192,884 cases due to missing data and invalid sample weights, 1,081,808 patients were included in the analysis. Following 1:1 propensity score matching, frailty and non-frailty were obtained in both metastatic (*n* = 99,017 per group) and non-metastatic groups (*n* = 418,435 per group).

Data from 1,274,692 patients with a diagnosis of CRC from the NIS database between 2005 and 2018 were extracted, as shown in Fig. 1. Subjects with missing data on outcomes of interest, sex, income, household income, primary payer, admission type, hospital bed size, or race/ethnicity (I = 187,605) were excluded. Data lacking sample weights for deriving national estimates were also excluded (I = 5279). Finally, data from 1,081,808 patients were retrieved, including 195,609 patients with metastatic CRC and 886,199 with non-metastatic disease. Within each CRC subgroup (metastatic and non-metastatic), PSM was performed to match frail and non-frail patients in a 1:1 ratio. After matching, 99,017 patients with metastatic disease (representing 484,476 hospitalised patients in the entire US after weighting) and 418,435 patients with non-metastatic disease (representing 1,023,436 hospitalised patients in the entire US after weighting) were included as the primary analytic sample. (Fig. 1).

### Characteristics of patients admitted with CRC

Before PSM, patients’ demographic and clinical characteristics, hospital information, and clinical outcomes are summarised in Supplementary Tables 2 and 3. In the metastatic CRC group, the mean age was 72.9 years, and 53.1% were male. In the non-metastatic CRC group, the mean age was 77.3 years, and 51.4% were females (Supplementary Table 2). In both metastatic and non-metastatic CRC groups, patients with frailty had higher percentages of adverse clinical outcomes compared to those without frailty (Supplementary Table 3).

Supplementary Table 4 shows that the HFRS increased with age in both metastatic and non-metastatic CRC patients before PSM.

After PSM, covariate balance was achieved (all SMDs < 0.1), except for hospital region in the non-metastatic CRC group (Table 1).

**Table 1:** Demographic and clinical characteristics of older CRC patients with/without metastases stratified by frailty status, after PSM

Characteristics	Metastatic				Non-Metastatic			
	Total (*n* = 99,017)	Frailty		SMD	Total (*n* = 418,435)	Frailty		SMD
		Yes (*n* = 49,368)	No (*n* = 49,649)			Yes (*n* = 209,089)	No (*n* = 209,346)	
Hospital frailty risk score	7.00 ± 0.03	11.56 ± 0.04	2.46 ± 0.01	**1.326**	7.28 ± 0.03	12.11 ± 0.03	2.46 ± 0.00	**1.083**
Demography								
Age, years	72.61 ± 0.04	72.74 ± 0.05	72.49 ± 0.05	0.006	76.91 ± 0.03	77.16 ± 0.04	76.67 ± 0.03	0.014
60–69	43,853 (41.8)	21,968 (41.8)	21,885 (41.8)	0.000	104,309 (24.5)	52,078 (24.4)	52,231 (24.6)	0.005
70–79	36,340 (34.6)	18,129 (34.6)	18,211 (34.7)		143,293 (33.6)	71,524 (33.6)	71,769 (33.7)	
80+	24,793 (23.6)	12,396 (23.6)	12,397 (23.5)		178,264 (41.9)	89,331 (42.0)	88,933 (41.8)	
Sex				0.056				0.068
Male	54,469 (51.9)	26,482 (50.5)	27,987 (53.3)		202,530 (47.6)	97,669 (45.9)	104,861 (49.3)	
Female	50,517 (48.1)	26,011 (49.5)	24,506 (46.7)		223,336 (52.4)	115,264 (54.1)	108,072 (50.7)	
Race/ethnicity				0.031				0.069
White	78,972 (75.2)	39,708 (75.7)	39,264 (74.8)		347,665 (81.6)	176,228 (82.8)	171,437 (80.5)	
Black	12,843 (12.2)	6486 (12.3)	6357 (12.1)		37,107 (8.7)	18,264 (8.6)	18,843 (8.9)	
Hispanic	7271 (6.9)	3480 (6.6)	3791 (7.2)		22,971 (5.4)	10,269 (4.8)	12,702 (6.0)	
Other/unknown	5900 (5.6)	2819 (5.4)	3081 (5.9)		18,123 (4.3)	8172 (3.8)	9951 (4.7)	
Household income				0.041				0.039
Q1	27,762 (26.5)	13,561 (25.9)	14,201 (27.1)		108,673 (25.6)	52,826 (24.8)	55,847 (26.3)	
Q2	26,501 (25.2)	12,990 (24.7)	13,511 (25.7)		111,824 (26.2)	55,655 (26.1)	56,169 (26.3)	
Q3	25,460 (24.2)	13,006 (24.8)	12,454 (23.7)		104,169 (24.4)	52,779 (24.8)	51,390 (24.1)	
Q4	25,263 (24.1)	12,936 (24.7)	12,327 (23.5)		101,200 (23.8)	51,673 (24.3)	49,527 (23.2)	
Primary payer				0.010				0.003
Medicare/Medicaid	82,147 (78.2)	41,103 (78.3)	41,044 (78.2)		366,013 (86.0)	182,968 (86.0)	183,045 (86.0)	
Private including HMO	19,237 (18.3)	9597 (18.3)	9640 (18.4)		50,386 (11.8)	25,271 (11.8)	25,115 (11.8)	
Self-pay/nocharge/other	3602 (3.4)	1793 (3.4)	1809 (3.5)		9467 (2.2)	4694 (2.2)	4773 (2.2)	
Admission type				0.003				0.008
Elective	26,010 (24.7)	13,037 (24.7)	12,973 (24.6)		122,386 (28.6)	60,860 (28.4)	61,526 (28.8)	
Emergent	78,976 (75.3)	39,456 (75.3)	39,520 (75.4)		303,480 (71.4)	152,073 (71.6)	151,407 (71.2)	
Year of admission				0.009				0.019
2005-2009	38,643 (36.0)	19,351 (36.1)	19,292 (35.9)		153,146 (35.2)	77,070 (35.4)	76,076 (34.9)	
2010-2014	31,065 (29.6)	15,510 (29.6)	15,555 (29.7)		128,257 (30.2)	64,496 (30.4)	63,761 (30.0)	
2015-2018	35,278 (34.3)	17,632 (34.3)	17,646 (34.4)		144,463 (34.7)	71,367 (34.2)	73,096 (35.1)	
Obesity	5162 (5.0)	2542 (4.9)	2620 (5.1)	0.012	25,517 (6.1)	11,770 (5.6)	13,747 (6.6)	0.042
Active tobacco use	22,615 (21.7)	11,323 (21.8)	11,292 (21.7)	0.006	95,872 (22.7)	48,013 (22.7)	47,859 (22.7)	0.001
**Major comorbidities**								
Ischaemic heart disease	17,730 (16.9)	8818 (16.8)	8912 (17.0)	0.004	128,871 (30.3)	64,503 (30.3)	64,368 (30.3)	0.001
Congestive heart failure	8467 (8.1)	4220 (8.1)	4247 (8.1)	0.004	58,427 (13.8)	29,167 (13.8)	29,260 (13.8)	0.001
Diabetes	23,735 (22.7)	11,842 (22.6)	11,893 (22.7)	0.009	116,455 (27.4)	57,785 (27.2)	58,670 (27.6)	0.010
Cerebrovascular disease	2048 (2.0)	1060 (2.0)	988 (1.9)	0.009	16,364 (3.8)	8416 (4.0)	7948 (3.7)	0.011
Chronic pulmonary disease	17,148 (16.4)	8594 (16.4)	8554 (16.3)	0.004	102,445 (24.1)	51,460 (24.2)	50,985 (24.0)	0.006
Severe liver disease	2103 (2.0)	894 (1.7)	1209 (2.3)	0.045	2982 (0.7)	1181 (0.6)	1801 (0.9)	0.035
Moderate or severe renal disease	6624 (6.4)	3270 (6.3)	3354 (6.4)	0.009	47,626 (11.3)	23,786 (11.3)	23,840 (11.3)	0.001
Systemic connective tissue disorders	11,757 (11.2)	5935 (11.3)	5822 (11.0)	0.012	51,643 (12.1)	26,158 (12.3)	25,485 (11.9)	0.010
**CCI**				0.013				0.001
0–1	82,353 (78.3)	41,259 (78.5)	41,094 (78.2)		284,093 (66.5)	142,075 (66.5)	142,018 (66.5)	
2–3	17,772 (17.0)	8827 (16.9)	8945 (17.1)		106,078 (25.0)	52,970 (25.0)	53,108 (25.0)	
4+	4861 (4.7)	2407 (4.6)	2454 (4.7)		35,695 (8.5)	17,888 (8.5)	17,807 (8.5)	
**Hospital bed size**				0.014				0.033
Small	15,049 (14.1)	7586 (14.2)	7463 (14.0)		72,505 (16.8)	37,255 (17.3)	35,250 (16.3)	
Medium	26,891 (25.8)	13,585 (26.1)	13,306 (25.5)		114,860 (27.1)	58,053 (27.4)	56,807 (26.9)	
Large	63,046 (60.1)	31,322 (59.7)	31,724 (60.5)		238501 (56.1)	117,625 (55.3)	120,876 (56.8)	
**Location/teaching status**				0.024				0.011
Rural	10331 (9.9)	5104 (9.8)	5227 (10.0)		51,104 (12.0)	25,796 (12.1)	25,308 (11.9)	
Urban nonteaching	36,023 (34.0)	18,253 (34.5)	17,770 (33.5)		159,057 (37.1)	79,671 (37.3)	79,386 (37.0)	
Urban teaching	58,632 (56.1)	29,136 (55.7)	294,96 (56.5)		215,705 (50.9)	107,466 (50.6)	108,239 (51.1)	
**Hospital region**				0.002				**0.225**
Northeast	28,018 (27.0)	14,030 (27.1)	13,988 (26.8)		121,366 (28.8)	64,114 (30.5)	57,252 (27.0)	
Midwest	19,910 (19.0)	9996 (19.1)	9914 (19.0)		93,837 (22.1)	52,052 (24.5)	41,785 (19.7)	
South	40,198 (38.1)	20,066 (38.0)	20,132 (38.2)		157,765 (36.8)	77,319 (36.0)	80,446 (37.7)	
West	16,860 (15.9)	8401 (15.8)	8459 (16.0)		52,898 (12.3)	19,448 (9.0)	33,450 (15.6)	
**Severity of illness subclass**				0.014				0.026
No class specified	16 (0.0)	7 (0.0)	9 (0.0)		50 (0.0)	23 (0.0)	27 (0.0)	
Minor loss of function	2333 (2.2)	1200 (2.3)	1133 (2.1)		72,041 (16.9)	36,928 (17.3)	35,113 (16.4)	
Moderate loss of function	44,220 (42.0)	22,028 (41.8)	22,192 (42.1)		232,150 (54.5)	114,882 (53.9)	117,268 (55.0)	
Major loss of function	51,419 (49.1)	25,757 (49.2)	25,662 (49.0)		107,143 (25.2)	53,824 (25.4)	53,319 (25.1)	
Extreme loss of function	6998 (6.7)	3501 (6.7)	3497 (6.7)		14,482 (3.4)	7276 (3.4)	7206 (3.4)	
**CRC-directed surgery**				0.006				0.003
No CRC-directed surgery	89,532 (85.3)	44,820 (85.4)	44,712 (85.2)		358,947 (84.3)	179,587 (84.4)	179,360 (84.3)	
Open surgery	10,967 (10.4)	5448 (10.3)	5519 (10.5)		46,004 (10.7)	22,929 (10.7)	23,075 (10.8)	
Laparoscopic/minimally invasive surgery	2128 (2.0)	1070 (2.1)	1058 (2.0)		20,792 (4.9)	10,357 (4.9)	10,435 (4.9)	
Liver metastasectomy	2359 (2.2)	1155 (2.2)	1204 (2.3)		-	-	-	

Categorical variables are presented as unweighted counts (weighted percentages).

Continuous variables are presented as mean ± SE.

SMD ≥ 0.1 is shown in bold.

*CCI* Charlson’s Comorbidity Index, *CRC* colorectal cancer, *PSM* propensity score matching, *HMO* Health Maintenance Organisation, *SMD* standardised mean difference.

Table 2 presents outcomes of patients after PSM, stratified by metastatic status and frailty status. Patients with frailty tended to have higher in-hospital mortality, greater proportions of prolonged LOS and discharge to long-term care facilities. (Table 2)

**Table 2:** Outcomes of older patients admitted with CRC, after PSM

Characteristic	Metastatic				Non-metastatic			
	Total (*n* = 105,576)	Frailty		SMD	Total (*n* = 432,198)	Frailty		SMD
		Yes (*n* = 52,788)	No (*n* = 52,788)			Yes (*n* = 216,009)	No (*n* = 216,009)	
**Outcomes**								
In-hospital mortality	5969 (5.7)	3125 (5.9)	2844 (5.4)	0.023	7431 (1.7)	3844 (1.8)	3587 (1.7)	0.009
Prolonged LOS ^a, b^	21,839 (22.0)	11,979 (24.2)	9860 (19.8)	**0.108**	80,488 (19.2)	45,358 (21.7)	35,130 (16.7)	**0.127**
Discharge to long-term care facilities ^a^	20,137 (20.4)	12,059 (24.5)	8078 (16.3)	**0.206**	91,455 (21.9)	59,135 (28.4)	32,320 (15.5)	**0.315**
Total hospital costs, USD	48,271.60 ± 349.85	49,702.16 ± 416.99	46,848.07 ± 385.91	0.013	42,258.91 ± 216.56	42,305.93 ± 246.73	42,211.85 ± 238.21	0.000

Continuous variables are presented as mean ± SE.

Categorical variables are presented as unweighted counts (weighted percentages).

SMD ≥ 0.1 is shown in bold.

*LOS* length of hospital stay, *CRC* colorectal cancer, *PSM* propensity score matching, *SMD* standardised mean difference.

^a^ Excluded patients who died in the hospital.

^b^ LOS > 75th percentile (metastatic subgroup: 8 days; non-metastatic subgroup: 7 days).

### Impact of frailty on clinical outcomes in patients admitted with CRC

The associations between frailty and outcomes of CRC admission are shown in Table 3. Among patients with metastatic disease, frailty was significantly associated with increased odds of mortality (odds ratio [OR] = 1.10; 95% CI:1.05–1.17), prolonged LOS (OR = 1.30; 95% CI:1.26–1.34), and discharge to long-term care facilities (OR = 1.67; 95% CI: 1.62–1.73). Frailty was also significantly associated with higher total hospital costs in the metastatic group, with an increase of $3750 USD (95% CI: 2940–4560) compared to patients without frailty.

**Table 3:** Impact of frailty (vs. non-frailty) on clinical outcomes in older patients admitted with CRC, after PSM.

Outcomes	CRC			
	Metastatic		Non-metastatic	
	OR (95% CI)	P-value	aOR (95% CI) ^c^	P-value
In-hospital mortality	**1.10 (1.05–1.17)**	**<0.001**	**1.05 (1.00–1.10)**	**0.044**
Prolonged LOS ^a, b^	**1.30 (1.26–1.34)**	**<0.001**	**1.37 (1.34–1.39)**	**<0.001**
Discharge to long-term care facilities ^a^	**1.67 (1.62–1.73)**	**<0.001**	**2.10 (2.07–2.14)**	**<0.001**
	Beta (95% CI)	P-value	Beta (95% CI)^c^	P-value
Total hospital costs, thousand USD	**3.75 (2.94–4.56)**	**<0.001**	**1.92 (1.48–2.36)**	**<0.001**

*OR* odds ratio, *CI* confidence interval, *aOR* adjusted odds ratio, *LOS* length of hospital stay, *CRC* colorectal cancer, *PSM* propensity score matching.

P-values < 0.05 are shown in bold.

^a^Excluded patients who died in the hospital.

^b^LOS >75th percentile (metastatic subgroup: 8 days; non-metastatic subgroup: 7 days).

^c^Adjusted for the variables with a SMD ≥ 0.1 after PSM (i.e., hospital region).

Similar results were observed among patients with non-metastatic CRC. Frailty was significantly associated with increased odds for mortality (adjusted OR [aOR] = 1.05; 95% CI: 1.00–1.10), prolonged LOS (aOR = 1.37; 95% CI: 1.34–1.39), and discharge to long-term care facilities (aOR = 2.10; 95% CI: 2.07–2.14). Frailty was also significantly associated with higher total hospital costs in the non-metastatic group, with an increase of $1920 USD (95% CI: 1480–2360) compared to patients without frailty. (Table 3)

### Impact of frailty on mortality in patients admitted with CRC, stratified by age, sex, and race/ethnicity

**Fig. 2:**
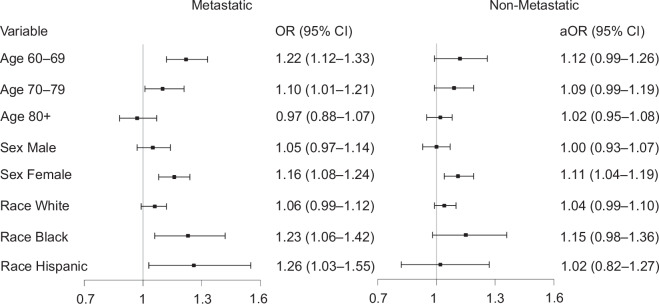
Impact of frailty (vs. non-frailty) on in-hospital mortality in patients admitted for metastatic or non-metastatic CRC, stratified by age, sex, and race/ethnicity. OR odds ratio, CI confidence interval, CRC colorectal cancer

Figure 2 illustrates the associations between frailty and in-hospital mortality, stratified by age, sex, and race/ethnicity separately for the metastatic and non-metastatic CRC groups. For metastatic CRC, across age subgroups, frailty was most strongly associated with in-hospital mortality among patients aged 60–69 years (OR = 1.22), with the effect diminishing with age and becoming non-significant in those aged 80 or older. For metastatic CRC, frailty was associated with increased in-hospital mortality risk among female. (Fig. 2)

### Impact of frailty on prolonged LOS in patients admitted with CRC, stratified by age, sex, and race/ethnicity

**Fig. 3:**
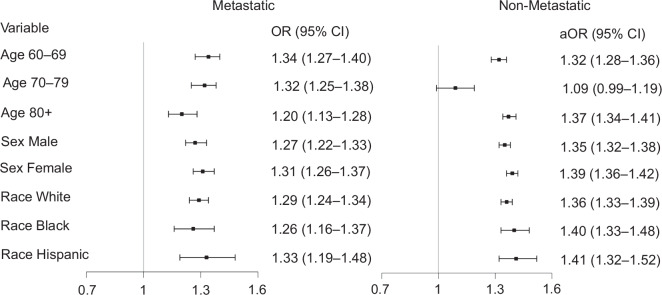
Impact of frailty (vs. non-frailty) on prolonged LOS ^a, b^ in patients admitted for metastatic or non-metastatic CRC, stratified by age, sex, and race/ethnicity. LOS, length of stay; OR, odds ratio; CI, confidence interval; CRC, colorectal cancer. ^a^ Excluded patients who died in the hospital. ^b^ LOS >75th percentile (metastatic: 8 days; non-metastatic: 6 days).

Figure 3 shows that frailty was significantly associated with prolonged LOS across all age, sex, and race/ethnicity subgroups in the metastatic CRC group. In the non-metastatic CRC group, a similar pattern was observed, with consistent associations between frailty and prolonged LOS across nearly all subgroups. (Fig. 3)

## Discussion

This study used a large US database to examine the impact of frailty, assessed by the HFRS, on clinical outcomes in older adults with CRC. Frailty was independently associated with higher mortality, longer hospital stays, and discharge to long-term care facilities regardless of metastasis status, and was also associated with increased total hospital costs in patients with or without metastatic disease. Subgroup analyses showed that the strength and significance of the associations varied across age, sex, and race/ethnicity. In metastatic CRC, frailty had the strongest association with mortality among patients aged 60–69 years, and remained significant among females, Black patients, and Hispanic patients. In non-metastatic CRC, the frailty–mortality association was overall weaker, with statistical significance observed mainly in selected subgroups such as females.

The concept of frailty is increasingly recognised as an important issue in clinical practice, especially in cancer patients undergoing surgery, chemotherapy, or radiotherapy. Both cancer itself and the treatments implemented can be important additional stressors that deplete patients’ physiological reserves, so frailty rates are particularly high in older cancer patients, with more than half being disabled by it [20]. Although age itself also affects the management of CRC, frailty has previously been shown to be associated with higher mortality and morbidity in these patients, whereas age itself appeared not [21].

Our stratified analyses suggested that frailty was associated with an increased risk of in-hospital mortality in both metastatic and non-metastatic CRC across age, sex, and race/ethnicity subgroups. Consistent with our findings, prior meta-analyses have demonstrated that frailty at admission is a strong predictor of poorer survival in patients with CRC, independent of sociodemographic characteristics and tumour features [15, 22]. These studies further suggested that early identification of frailty through comprehensive assessment may help optimise clinical management and improve outcomes in older patients with CRC [22]. In surgical cohorts, frailty has been shown to be common and to carry important prognostic value in CRC resection, including associations with delayed postoperative recovery and increased complication risk [23, 24]. Despite these observations, the underlying mechanisms linking frailty to adverse clinical outcomes in CRC remain incompletely understood and warrant further investigation.

Notably, the association between frailty and in-hospital mortality appeared stronger in patients aged 60–69 and weaker in those aged ≥80. This pattern warrants further explanation. First, a ceiling effect may be present—advanced age alone confers a high baseline mortality risk, which may diminish the relative impact of frailty. Second, differences in care intensity may contribute, as patients aged ≥80 are often less likely to receive aggressive interventions regardless of frailty status, potentially blunting differences in outcomes. Conversely, frail individuals aged 60–69 may represent a subgroup with unexpectedly poor physiologic reserve relative to their age, making the impact of frailty more pronounced.

Further, several randomised controlled trials have demonstrated that geriatric assessment (GA)-guided interventions can improve outcomes in older adults with cancer, particularly among those identified as vulnerable through GA screening. For example, the GAP70+ trial enroled older patients with advanced cancer who had at least one impaired GA domain and showed that providing oncologists with GA summaries and tailored recommendations significantly reduced grade 3–5 treatment-related toxicity without compromising overall survival [25]. Similarly, the GAIN trial implemented multidisciplinary interventions for patients with abnormal GA findings and observed a 10% absolute reduction in grade 3 or higher chemotherapy-related toxic effects [26]. The INTEGERATE trial, which integrated GA into routine care for all older patients initiating systemic anticancer therapy regardless of screening status, demonstrated improvements in quality of life and reductions in unplanned hospital admissions [27]. While these studies emphasise the clinical value of structured GA interventions, our study adds a complementary, population-level perspective by using the Hospital Frailty Risk Score (HFRS) to examine the impact of frailty in a large cohort of older adults hospitalised for colorectal cancer. Although frailty is a core domain within GA, the two are not synonymous—GA provides a comprehensive multidimensional assessment of aging-related vulnerabilities, whereas frailty represents a focused measure of physiological reserve and vulnerability.

Importantly, unlike the above cited trials that enroled selected patients eligible for systemic therapy, our study reflects real-world clinical practice by evaluating the impact of frailty—as measured by the HFRS—in a large, administrative dataset with both metastatic and non-metastatic CRC. Based on our analytic results, we agree and recommend that, given older adults comprise the majority of the CRC population, systematic frailty screening should be implemented to more accurately identify high-risk patients, guide individualised treatment decisions, and improve risk stratification and clinical decision-making in inpatient care. Specifically, tools such as the HFRS can support inpatient risk stratification at scale, while comprehensive GA may be better suited for outpatient oncology settings. Together, these findings underscore the value of integrating frailty assessment into routine cancer care to inform care planning and optimise outcomes. Further studies may explore how claims-based frailty metrics can be prospectively implemented to trigger targeted geriatric support during hospitalisation.

### Strengths and limitations

The study utilised a large, nationally representative dataset, enhancing the generalisability of the findings. Furthermore, PSM was applied to control for confounding variables, leading to more robust estimates of the association between frailty and surgical outcomes. However, this study has several limitations. First, its retrospective design may introduce selection bias and limit the ability to draw robust causal inferences. The NIS is a discharge-level, not patient-level, database; unique identifiers are not available, and repeated admissions for the same individual cannot be linked. Potential repeated hospitalisations may slightly overrepresent certain patients. However, the sampling design makes the likelihood of duplicate capture low and unlikely to meaningfully influence population-level estimates. Second, the NIS dataset is based on hospitalised patients in the US, the findings may not be generalisable to other countries with different healthcare systems and patient populations. The identification of diseases relied on claims codes, and the possibility of coding errors exists when using the ICD coding system to confirm inpatient diagnoses. The HFRS was calculated using ICD codes recorded during the index hospitalisation, as look-back data are not available in the NIS. This may affect frailty ascertainment because individuals with limited prior healthcare contact may not have accumulated frailty-related diagnostic codes, potentially leading to misclassification as non-frail and attenuating the observed associations between frailty and outcomes. Third, hospitalisations were included if CRC appeared in any diagnosis position in the NIS records, ensuring that the full spectrum of admissions among patients with CRC was captured. However, this approach may result in clinical heterogeneity, as admissions in older patients with CRC could have been triggered by cancer-associated complications or acute non-cancer conditions (e.g., infection, cardiovascular events). Correspondingly, a large proportion of patients did not undergo CRC-directed surgery during the index admission, and the population largely comprised acutely hospitalised patients rather than individuals admitted for elective oncologic surgery. As a result, generalisability to elective surgical oncology populations and oncology-focused inpatient settings may be lower than expected, although the study sample reflects real-world inpatient care. Fourth, information on prior oncologic treatments—including previous surgery, chemotherapy, radiation, targeted agents, treatment intent (e.g., curative oncologic therapy versus palliative or symptom-directed management), and the time window of CRC diagnosis—is not available in the NIS. Chemotherapy and radiotherapy codes recorded during the index hospitalisation are known to be substantially undercoded and unvalidated in administrative datasets, and therefore were not used to avoid misclassification. Finally, the NIS does not provide post-discharge follow-up, precluding the evaluation of long-term outcomes such as late mortality, readmissions, or functional decline, and it does not capture patients’ family history or genetic background, which may be relevant to both CRC and frailty.

## Conclusions

Frailty assessed by HFRS predicts poorer clinical outcomes for older patients aged ≥60 years with CRC, including increased in-hospital mortality, prolonged LOS, discharge to long-term care facilities, regardless of metastasis status. The association between frailty and in-hospital mortality appears to be strongest among patients aged 60–69 years with metastatic disease. These findings lay the foundation for risk stratification and for developing treatment strategies and targeted interventions for older patients with CRC and frailty.

## Supplementary information

aj-checklist_BJC-A3351484R2

Supplementary Table 1-7

## Data Availability

Data are available from the corresponding author upon reasonable request.

## References

[1] Fadlallah H, El Masri J, Fakhereddine H, Youssef J, Chemaly C, Doughan S, et al. Colorectal cancer: recent advances in management and treatment. World J Clin Oncol. 2024;15:1136–56. 39351451 10.5306/wjco.v15.i9.1136 PMC11438855

[2] Breekveldt ECH, Lansdorp-Vogelaar I, Toes-Zoutendijk E, Spaander MCW, Van Vuuren AJ, Van Kemenade FJ, et al. Colorectal cancer incidence, mortality, tumour characteristics, and treatment before and after introduction of the faecal immunochemical testing-based screening programme in the Netherlands: a population-based study. Lancet Gastroenterol Hepatol. 2022;7:60–8. 34822762 10.1016/S2468-1253(21)00368-X

[3] Dekker E, Tanis PJ, Vleugels JLA, Kasi PM, Wallace MB. Colorectal cancer. Lancet. 2019;394:1467–80. 31631858 10.1016/S0140-6736(19)32319-0

[4] Biller LH, Schrag D. Diagnosis and treatment of metastatic colorectal cancer. JAMA. 2021;325:669. 33591350 10.1001/jama.2021.0106

[5] Alswealmeen W, Sadri L, Perrotti G, Heilman J, Bakshi K, Kim SY, et al. Colorectal cancer screening in the elderly: is age just a number? Clin Colorectal Cancer. 2022;21:e113–e6. 34955377 10.1016/j.clcc.2021.11.011

[6] Hoogendijk EO, Afilalo J, Ensrud KE, Kowal P, Onder G, Fried LP. Frailty: implications for clinical practice and public health. Lancet. 2019;394:1365–75. 31609228 10.1016/S0140-6736(19)31786-6

[7] Davey MG, Joyce WP. Impact of frailty on oncological outcomes in patients undergoing surgery for colorectal cancer – A systematic review and meta-analysis. Surgeon. 2023;21:173–80. 35792005 10.1016/j.surge.2022.06.001

[8] Leng S., Chen X., Mao G. Frailty syndrome: an overview. Clin Interven Aging. 2014;9:433–41. 10.2147/CIA.S45300 PMC3964027 24672230

[9] Fried LP, Cohen AA, Xue Q-L, Walston J, Bandeen-Roche K, Varadhan R. The physical frailty syndrome as a transition from homeostatic symphony to cacophony. Nat Aging. 2021;1:36–46. 34476409 10.1038/s43587-020-00017-z PMC8409463

[10] Song X, Mitnitski A, Rockwood K. Prevalence and 10-Year outcomes of frailty in older adults in relation to deficit accumulation. J Am Geriatr Soc. 2010;58:681–7. 20345864 10.1111/j.1532-5415.2010.02764.x

[11] Gilbert T, Neuburger J, Kraindler J, Keeble E, Smith P, Ariti C, et al. Development and validation of a Hospital Frailty Risk Score focusing on older people in acute care settings using electronic hospital records: an observational study. Lancet. 2018;391:1775–82. 29706364 10.1016/S0140-6736(18)30668-8 PMC5946808

[12] Sinclair De Frías J, Olivero L, Gabela A, Jaen D, Menser T, Moreno Franco P. Frailty predicts adverse outcomes in older patients with pulmonary embolism. Geriatr Gerontol Int. 2024;24:924–9. 39143935 10.1111/ggi.14961

[13] Abugroun A, Daoud H, Hallak O, Abdel-Rahman ME, Klein LW. Frailty predicts adverse outcomes in older patients undergoing transcatheter aortic valve replacement (TAVR): from the National Inpatient Sample. Cardiovasc Revascularization Med. 2022;34:56–60. 10.1016/j.carrev.2021.02.004 33632638

[14] Siddiqui E, Banco D, Berger JS, Smilowitz NR. Frailty assessment and perioperative major adverse cardiovascular events after noncardiac surgery. Am J Med. 2023;136:372–9.e5. 36657557 10.1016/j.amjmed.2022.12.033 PMC10038881

[15] Michaud Maturana M, English WJ, Nandakumar M, Li Chen J, Dvorkin L. The impact of frailty on clinical outcomes in colorectal cancer surgery: a systematic literature review. ANZ J Surg. 2021;91:2322–9. 34013571 10.1111/ans.16941

[16] Powell-Cope G., Nelson A. L., Patterson E. S. Advances in patient safety. In: Hughes R. G., editor. Patient safety and quality: an evidence-based handbook for nurses. Rockville (MD): Agency for Healthcare Research and Quality (US); 2008. 21328752

[17] Nghiem S, Afoakwah C, Scuffham P, Byrnes J. Hospital frailty risk score and adverse health outcomes: evidence from longitudinal record linkage cardiac data. Age Ageing. 2021;50:1778–84. 33989395 10.1093/ageing/afab073

[18] Huan-Tze L., Yun-Ru L., Kuan-Der L., Huey-En T. Frailty in chronic myeloid leukemia: evidence from 2016–2018 Nationwide Inpatient Sample of the US. BMC Geriatr. 2023;23:334. 10.1186/s12877-023-03962-7 PMC10228104 37254068

[19] Glasheen WP, Cordier T, Gumpina R, Haugh GS, Davis J, Renda A. Charlson comorbidity index: ICD-9 Update and ICD-10 translation. Am health drug benefits. 2019;12:188–97. 31428236 PMC6684052

[20] O’Donovan A, Leech M. Personalised treatment for older adults with cancer: the role of frailty assessment. Tech Innov Patient Support Radiat Oncol. 2020;16:30–8. 33102819 10.1016/j.tipsro.2020.09.001 PMC7568178

[21] Ørum M, Gregersen M, Jensen K, Meldgaard P, Damsgaard EMS. Frailty status but not age predicts complications in elderly cancer patients: a follow-up study. Acta Oncol. 2018;57:1458–66. 30280625 10.1080/0284186X.2018.1489144

[22] Boakye D, Rillmann B, Walter V, Jansen L, Hoffmeister M, Brenner H. Impact of comorbidity and frailty on prognosis in colorectal cancer patients: a systematic review and meta-analysis. Cancer Treat Rev. 2018;64:30–9. 29459248 10.1016/j.ctrv.2018.02.003

[23] McGovern J., Dolan RD, Horgan PG, Laird BJ, McMillan DC. The prevalence and prognostic value of frailty screening measures in patients undergoing surgery for colorectal cancer: observations from a systematic review. BMC Geriatr. 2022;22:260. 10.1186/s12877-022-02928-5 PMC8962494 35351011

[24] Gong W, Qi X. Association of frailty with delayed recovery of gastrointestinal function after elective colorectal cancer resections. J Investig Surg. 2020;33:544–50. 30430890 10.1080/08941939.2018.1524528

[25] Mohile SG, Mohamed MR, Xu H, Culakova E, Loh KP, Magnuson A, et al. Evaluation of geriatric assessment and management on the toxic effects of cancer treatment (GAP70+): a cluster-randomised study. Lancet. 2021;398:1894–904. 34741815 10.1016/S0140-6736(21)01789-X PMC8647163

[26] Li D, Sun C-L, Kim H, Soto-Perez-De-Celis E, Chung V, Koczywas M, et al. Geriatric Assessment–Driven Intervention (GAIN) on chemotherapy-related toxic effects in older adults with cancer. JAMA Oncol. 2021;7:e214158. 34591080 10.1001/jamaoncol.2021.4158 PMC8485211

[27] Soo WK, King MT, Pope A, Parente P, Dārziņš P, Davis ID. Integrated Geriatric Assessment and Treatment Effectiveness (INTEGERATE) in older people with cancer starting systemic anticancer treatment in Australia: a multicentre, open-label, randomised controlled trial. Lancet Healthy Longev. 2022;3:e617–e27. 36102776 10.1016/S2666-7568(22)00169-6

