# Supplementary material for: The association between hospital frailty risk score and adverse inpatient outcomes in older adults with colorectal cancer
Source: Br J Cancer. 2026 May 6;135(3):418–26. doi: 10.1038/s41416-026-03385-2 (PMC13373189; doi:10.1038/s41416-026-03385-2)
Supplement: Supplementary file 2 — Supplementary Table 1-7 [file 41416_2026_3385_MOESM2_ESM.docx]

Supplementary Table 1. ICD codes used to define diagnosis and surgery.

|  | ICD-9-CM/ ICD-9-PCS | ICD-10-CM/ ICD-10-PCS |
| --- | --- | --- |
| CRC | 153, 154.0, 154.1, V10.05, V10.06 | C18-C20, Z85.03, Z85.04 |
| Metastatic | 197-198 | C78, C79 |
| Obesity | 278.0, 278.1, V85.21-V85.45 | E66, Z68.25-Z68.45 |
| Active tobacco use | 305.1, V15.82, 989.84 | Z71.6, Z72.0, Z86.43, Z87.891, F17, O99.33, T65.2 |
| Ischemic heart disease | 410–414 | I25 |
| Congestive heart failure |  | I09.81, I11.0, I13.0, I13.2, I50 |
| Diabetes |  | E10-E14 |
| Cerebrovascular disease | 362.34, 430-438 | G45, G46, H34.0, I60-I69 |
| Chronic pulmonary disease | 416.8, 416.9, 490-505, 506.4, 508.1, 508.8 | I27.8, I27.9, J40 -J47, J60-J67, J68.4, J70.1, J70.3 |
| Severe liver disease | 456.0–456.2, 572.2–572.8 | I85.0, I85.9, I86.4, I98.2, K70.4, K71.1, K72.1, K72.9, K76.5, K76.6, K76.7 |
| Moderate or severe renal disease | 403.01, 403.11, 403.91, 404.02, 404.03, 404.12, 404.13, 404.92, 404.93, 582, 583.0-583.7, 585, 586, 588.0, V42.0, V45.1, V56 | I12.0, I13.1, N03.2-N03.7,  N05.2-N05.7, N18, N19, N25.0,  Z49.0-Z49.2, Z94.0, Z99.2 |
| Systemic connective tissue disorders | 446.5, 710, 714.0-714.2, 714.8, 725 | M05, M06, M31.5, M30 -M36 |
| Open CRC resection | **PCS:** 45.71-45.76, 45.79, 45.82, 48.40, 48.41, 48.49, 48.50, 48.59, 48.61, 48.64, 48.65, 48.69 | **PCS:** 0DTE0ZZ/7ZZ, 0DTF0ZZ/7ZZ, 0DTG0ZZ/7ZZ, 0DTH0ZZ/7ZZ, 0DTJ0ZZ/7ZZ, 0DTK0ZZ/7ZZ, 0DTL0ZZ/7ZZ, 0DTM0ZZ/7ZZ, 0DTN0ZZ/7ZZ, 0DTP0ZZ/7ZZ, 0DBE0ZZ/7ZZ, 0DBF0ZZ/7ZZ, 0DBG0ZZ/7ZZ, 0DBH0ZZ/7ZZ, 0DBJ0ZZ/7ZZ, 0DBK0ZZ/7ZZ, 0DBL0ZZ/7ZZ, 0DBM0ZZ/7ZZ, 0DBN0ZZ/7ZZ, 0DBP0ZZ/7ZZ |
| Laparoscopic / minimally invasive CRC resection | **PCS:** 45.81, 48.42, 48.51, 17.31-17.36-17.39 | **PCS:** 0DTE4ZZ/8ZZ/FZZ, 0DTF4ZZ/8ZZ/FZZ, 0DTG4ZZ/8ZZ/FZZ, 0DTH4ZZ/8ZZ/FZZ, 0DTJ4ZZ/8ZZ/FZZ, 0DTK4ZZ/8ZZ/FZZ, 0DTL4ZZ/8ZZ/FZZ, 0DTM4ZZ/8ZZ/FZZ, 0DTN4ZZ/8ZZ/FZZ, 0DTP4ZZ/8ZZ/FZZ 0DBE3ZZ/4ZZ/8ZZ, 0DBF3ZZ/4ZZ/8ZZ, 0DBG3ZZ/4ZZ/8ZZ, 0DBH3ZZ/4ZZ/8ZZ, 0DBJ3ZZ/4ZZ/8ZZ, 0DBK3ZZ/4ZZ/8ZZ, 0DBL3ZZ/4ZZ/8ZZ, 0DBM3ZZ/4ZZ/8ZZ, 0DBN3ZZ/4ZZ/8ZZ, 0DBP3ZZ/4ZZ/8ZZ, 8E0W0CZ, 8E0W3CZ, 8E0W4CZ, 8E0W7CZ, 8E0W8CZ |
| Liver resection | **PCS:** 50.2-50.4 | **PCS:** 0FB00ZZ, 0FB03ZZ, 0FB04ZZ, 0F500ZZ, 0F503ZZ, 0F504ZZ, 0FT10ZZ, 0FT14ZZ, 0FT20ZZ, 0FT24ZZ  0FT00ZZ, 0FT04ZZ |

ICD, International Classification of Diseases; CM, Clinical Classification; PCS, Procedure Code System; CRC, colorectal cancer.

Supplementary Table 2. Characteristics of patients admitted with CRC, before PSM.

| **Characteristic** | **Metastatic** | | | |  | **Non-Metastatic** | | | |
| --- | --- | --- | --- | --- | --- | --- | --- | --- | --- |
|  | **Total**  **(n = 195,609)** | **Frailty** | | **SMD** |  | **Total**  **(n= 886,199)** | **Frailty** | | **SMD** |
|  |  | **Yes**  **(n = 122,095)** | **No**  **(n = 73,514)** |  |  |  | **Yes (n=575,517)** | **No**  **(n= 310,682)** |  |
| **Hospital frailty risk score** | 9.30 ± 0.03 | 13.54 ± 0.03 | 2.24 ± 0.01 | **1.096** |  | 10.47 ± 0.03 | 14.96 ± 0.02 | 2.17 ± 0.00 | **0.805** |
| **Demography** |  |  |  |  |  |  |  |  |  |
| Age, years | 72.94 ± 0.04 | 73.63 ± 0.04 | 71.79 ± 0.04 | 0.065 |  | 77.33 ± 0.02 | 78.53 ± 0.02 | 75.11 ± 0.03 | 0.097 |
| 60-69 | 78872 (40.4) | 45195 (37.0) | 33677 (45.9) | **0.203** |  | 208004 (23.5) | 110918 (19.3) | 97086 (31.3) | **0.357** |
| 70-79 | 68690 (35.1) | 43670 (35.8) | 25020 (34.0) |  |  | 286909 (32.4) | 178166 (31.0) | 108743 (35.0) |  |
| 80+ | 48047 (24.5) | 33230 (27.2) | 14817 (20.1) |  |  | 391286 (44.1) | 286433 (49.8) | 104853 (33.7) |  |
| Sex |  |  |  | 0.009 |  |  |  |  | 0.017 |
| Male | 103803 (53.1) | 64983 (53.3) | 38820 (52.8) |  |  | 430940 (48.6) | 278130 (48.3) | 152810 (49.2) |  |
| Female | 91806 (46.9) | 57112 (46.7) | 34694 (47.2) |  |  | 455259 (51.4) | 297387 (51.7) | 157872 (50.8) |  |
| Race/ethnicity |  |  |  | 0.056 |  |  |  |  | 0.077 |
| White | 145939 (74.6) | 91004 (74.5) | 54935 (74.7) |  |  | 716176 (80.8) | 468138 (81.3) | 248038 (79.8) |  |
| Black | 24954 (12.8) | 16269 (13.3) | 8685 (11.8) |  |  | 81330 (9.2) | 54374 (9.5) | 26956 (8.7) |  |
| Hispanic | 13436 (6.9) | 8027 (6.6) | 5409 (7.3) |  |  | 49190 (5.5) | 29380 (5.1) | 19810 (6.4) |  |
| Other/unknown | 11280 (5.8) | 6795 (5.6) | 4485 (6.1) |  |  | 39503 (4.5) | 23625 (4.1) | 15878 (5.1) |  |
| Household income |  |  |  | 0.019 |  |  |  |  | 0.015 |
| Q1 | 52325 (26.8) | 32630 (26.8) | 19695 (26.8) |  |  | 230631 (26.1) | 149626 (26.1) | 81005 (26.1) |  |
| Q2 | 48706 (24.9) | 30080 (24.6) | 18626 (25.3) |  |  | 230068 (26.0) | 148186 (25.8) | 81882 (26.3) |  |
| Q3 | 47314 (24.2) | 29814 (24.4) | 17500 (23.8) |  |  | 216207 (24.4) | 141106 (24.5) | 75101 (24.2) |  |
| Q4 | 47264 (24.2) | 29571 (24.2) | 17693 (24.1) |  |  | 209293 (23.6) | 136599 (23.7) | 72694 (23.4) |  |
| Primary payer |  |  |  | **0.148** |  |  |  |  | **0.196** |
| Medicare/Medicaid | 154609 (79.0) | 99294 (81.3) | 55315 (75.2) |  |  | 765313 (86.4) | 510868 (88.8) | 254445 (81.9) |  |
| Private including HMO | 34602 (17.7) | 19233 (15.8) | 15369 (20.9) |  |  | 102556 (11.6) | 54479 (9.4) | 48077 (15.5) |  |
| Self-pay/nocharge/other | 6398 (3.3) | 3568 (2.9) | 2830 (3.8) |  |  | 18330 (2.1) | 10170 (1.8) | 8160 (2.6) |  |
| Admission type |  |  |  | **0.381** |  |  |  |  | **0.442** |
| Elective | 47718 (24.3) | 22212 (18.1) | 25506 (34.6) |  |  | 244393 (27.5) | 118739 (20.6) | 125654 (40.4) |  |
| Emergent | 147891 (75.7) | 99883 (81.9) | 48008 (65.4) |  |  | 641806 (72.5) | 456778 (79.4) | 185028 (59.6) |  |
| Year of admission |  |  |  | **0.380** |  |  |  |  | **0.387** |
| 2005-2009 | 64429 (32.2) | 36808 (29.5) | 27621 (36.7) |  |  | 281398 (31.0) | 173339 (29.5) | 108059 (33.9) |  |
| 2010-2014 | 70743 (36.3) | 52208 (42.9) | 18535 (25.3) |  |  | 334616 (37.8) | 252397 (44.0) | 82219 (26.5) |  |
| 2015-2018 | 60437 (31.6) | 33079 (27.7) | 27358 (38.0) |  |  | 270185 (31.1) | 149781 (26.6) | 120404 (39.6) |  |
| Obesity | 13926 (7.2) | 10973 (9.0) | 2953 (4.1) | **0.200** |  | 81005 (9.2) | 64483 (11.2) | 16522 (5.4) | **0.212** |
| Active tobacco use | 50563 (26.0) | 36089 (29.7) | 14474 (19.9) | **0.229** |  | 233133 (26.5) | 169040 (29.5) | 64093 (20.8) | **0.201** |
| **Major comorbidities** |  |  |  |  |  |  |  |  |  |
| Ischemic heart disease | 36014 (18.4) | 24869 (20.4) | 11145 (15.2) | **0.137** |  | 275327 (31.1) | 194375 (33.8) | 80952 (26.1) | **0.170** |
| Congestive heart failure | 18002 (9.2) | 13116 (10.8) | 4886 (6.7) | **0.145** |  | 134665 (15.3) | 101532 (17.7) | 33133 (10.7) | **0.201** |
| Diabetes | 47285 (24.2) | 31456 (25.8) | 15829 (21.6) | **0.100** |  | 256276 (29.0) | 178534 (31.1) | 77742 (25.1) | **0.134** |
| Cerebrovascular disease | 7626 (3.9) | 6622 (5.4) | 1004 (1.4) | **0.225** |  | 69049 (7.8) | 60923 (10.6) | 8126 (2.6) | **0.325** |
| Chronic pulmonary disease | 34306 (17.6) | 23543 (19.3) | 10763 (14.7) | **0.124** |  | 219923 (24.8) | 156990 (27.3) | 62933 (20.3) | **0.166** |
| Severe liver disease | 4345 (2.2) | 3028 (2.5) | 1317 (1.8) | 0.048 |  | 6857 (0.8) | 4894 (0.9) | 1963 (0.6) | 0.025 |
| Moderate or severe renal disease | 23331 (12.0) | 19848 (16.3) | 3483 (4.8) | **0.384** |  | 166159 (18.9) | 141270 (24.7) | 24889 (8.1) | **0.461** |
| Systemic connective tissue disorders | 32344 (16.5) | 26304 (21.6) | 6040 (8.2) | **0.383** |  | 152675 (17.2) | 125943 (21.9) | 26732 (8.6) | **0.377** |
| **CCI** |  |  |  | **0.406** |  |  |  |  | **0.536** |
| 0-1 | 139877 (71.4) | 79399 (64.9) | 60478 (82.2) |  |  | 518357 (58.3) | 287476 (49.8) | 230881 (74.2) |  |
| 2-3 | 41705 (21.4) | 31297 (25.7) | 10408 (14.2) |  |  | 251349 (28.4) | 190448 (33.1) | 60901 (19.6) |  |
| 4+ | 14027 (7.2) | 11399 (9.4) | 2628 (3.6) |  |  | 116493 (13.3) | 97593 (17.1) | 18900 (6.2) |  |
| **Hospital bed size** |  |  |  | 0.020 |  |  |  |  | 0.024 |
| Small | 26976 (13.6) | 16649 (13.4) | 10327 (13.8) |  |  | 141038 (15.7) | 89846 (15.4) | 51192 (16.2) |  |
| Medium | 50011 (25.7) | 31578 (26.0) | 18433 (25.2) |  |  | 238425 (27.1) | 155441 (27.2) | 82984 (26.9) |  |
| Large | 118622 (60.7) | 73868 (60.6) | 44754 (60.9) |  |  | 506736 (57.3) | 330230 (57.5) | 176506 (56.9) |  |
| **Location/teaching status** |  |  |  | 0.070 |  |  |  |  | 0.076 |
| Rural | 18391 (9.4) | 11336 (9.3) | 7055 (9.6) |  |  | 101567 (11.5) | 65632 (11.5) | 35935 (11.6) |  |
| Urban nonteaching | 67447 (34.2) | 43564 (35.5) | 23883 (32.2) |  |  | 339472 (38.1) | 227405 (39.3) | 112067 (35.8) |  |
| Urban teaching | 109771 (56.3) | 67195 (55.2) | 42576 (58.2) |  |  | 445160 (50.4) | 282480 (49.2) | 162680 (52.7) |  |
| **Hospital region** |  |  |  | **0.100** |  |  |  |  | 0.090 |
| Northeast | 50157 (25.8) | 29577 (24.4) | 20580 (28.2) |  |  | 212342 (24.1) | 131645 (23.0) | 80697 (26.1) |  |
| Midwest | 37867 (19.4) | 24833 (20.4) | 13034 (17.8) |  |  | 188614 (21.4) | 128314 (22.4) | 60300 (19.5) |  |
| South | 73985 (37.7) | 46092 (37.7) | 27893 (37.8) |  |  | 336023 (37.9) | 217021 (37.7) | 119002 (38.2) |  |
| West | 33600 (17.0) | 21593 (17.5) | 12007 (16.2) |  |  | 149220 (16.7) | 98537 (16.9) | 50683 (16.2) |  |
| **Severity of illness subclass** |  |  |  | **0.808** |  |  |  |  | **0.883** |
| No class specified | 20 (0.01) | 10 (0.01) | 10 (0.01) |  |  | 74 (0.01) | 38 (0.01) | 36 (0.01) |  |
| Minor loss of function | 5477 (2.8) | 1356 (1.1) | 4121 (5.6) |  |  | 144541 (16.3) | 45329 (7.9) | 99212 (31.9) |  |
| Moderate loss of function | 68152 (34.8) | 28697 (23.4) | 39455 (53.6) |  |  | 369074 (41.6) | 218989 (38.0) | 150085 (48.3) |  |
| Major loss of function | 94572 (48.4) | 68141 (55.9) | 26431 (36.0) |  |  | 292625 (33.1) | 238483 (41.5) | 54142 (17.5) |  |
| Extreme loss of function | 27388 (14.0) | 23891 (19.6) | 3497 (4.8) |  |  | 79885 (9.0) | 72678 (12.7) | 7207 (2.3) |  |
| **CRC-directed surgery** |  |  |  | **0.222** |  |  |  |  | **0.349** |
| No CRC-directed surgery | 165498 (84.6) | 106826 (87.5) | 58672 (79.8) |  |  | 743001 (83.85) | 508511 (88.38) | 234490 (75.45) |  |
| Open surgery | 20946 (10.7) | 11292 (9.2) | 9654 (13.1) |  |  | 94945 (10.65) | 47840 (8.26) | 47105 (15.07) |  |
| Laparoscopic/minimally invasive surgery | 4548 (2.4) | 1970 (1.6) | 2578 (3.6) |  |  | 47974 (5.47) | 19012 (3.33) | 28962 (9.44) |  |
| Liver metastasectomy | 4617 (2.4) | 2007 (1.6) | 2610 (3.5) |  |  | - | - | - |  |

CCI, Charlson's Comorbidity Index; CRC, colorectal cancer; PSM, propensity score matching; HMO, Health Maintenance Organization; SMD, standardized mean difference.

Continuous variables are presented as mean ± SE. Categorical variables are presented as unweighted counts (weighted percentages).

SMD ≥ 0.1 is shown in bold.

Supplementary Table 3. Outcome of patients admitted with CRC, before PSM.

| **Characteristics** | **Metastatic** | | | |  | **Non-Metastatic** | | | |
| --- | --- | --- | --- | --- | --- | --- | --- | --- | --- |
|  | **Total**  **(n = 195,609)** | **Frailty** | | **SMD** |  | **Total**  **(n= 886,199)** | **Frailty** | | **SMD** |
|  |  | **Yes**  **(n = 122,095)** | **No**  **(n = 73,514)** |  |  |  | **Yes**  **(n=575,517)** | **No**  **(n= 310,682)** |  |
| **Outcomes** |  |  |  |  |  |  |  |  |  |
| In-hospital mortality | 14767 (7.5) | 11434 (9.4) | 3333 (4.5) | **0.191** |  | 27626 (3.1) | 23670 (4.1) | 3956 (1.3) | **0.176** |
| Prolonged LOS ^a, b^ | 36968 (20.4) | 27355 (24.7) | 9613 (13.6) | **0.314** |  | 189689 (22.1) | 145152 (26.3) | 44537 (14.5) | **0.314** |
| Discharge to long-term care facilities ^a^ | 41298 (22.9) | 31695 (28.7) | 9603 (13.7) | **0.374** |  | 221669 (25.8) | 182243 (33.1) | 39426 (12.9) | **0.494** |
| Total hospital cost, USD | 54799.25 ± 352.07 | 59852.90 ± 409.46 | 46399.99 ± 378.56 | 0.036 |  | 48273.00 ± 230.68 | 51130.73 ± 266.55 | 42978.47 ± 224.74 | 0.008 |

LOS, length of hospital stay; CRC, colorectal cancer; PSM, propensity score matching; SMD, standardized mean difference.

Continuous variables are presented as mean ± SE.

Categorical variables are presented as unweighted counts (weighted percentages).

SMD ≥ 0.1 is shown in bold.

^a^ Excluded patients who died in the hospital.

^b^ LOS >75th percentile (metastatic subgroup: 9 days; non-metastatic subgroup: 7 days).

Supplementary Table 4. Hospital Frailty Risk Score of older CRC patients with/without metastases across age categories, before PSM.

| Characteristics | Metastatic | | |  | Non-metastatic | | |
| --- | --- | --- | --- | --- | --- | --- | --- |
|  | Age, years | | |  | Age, years | | |
|  | 60-69 | 70-79 | 80+ |  | 60-69 | 70-79 | 80+ |
|  | n = 73,747 | n = 63,427 | n = 43,668 |  | n = 204,401 | n = 279,576 | n = 374,596 |
| Hospital Frailty Risk Score | 8.15 ± 0.04 | 9.26 ± 0.04 | 10.48 ± 0.05 |  | 8.02 ± 0.03 | 9.62 ± 0.03 | 12.13 ± 0.03 |

Continuous variables are presented as mean ± SE.

PSM, propensity score matching.

Supplementary Table 5. ICD-9 codes used to assess the Hospital Frailty Risk Score (HFRS).

| ICD-9 Condition | ICD-9-CM | Points |
| --- | --- | --- |
| Senile dementia, uncomplicated | 2900 | 7.1 |
| Dementia in conditions classified elsewhere | 2941 | 7.1 |
| Hemiplegia | 342 | 4.4 |
| Alzheimer's disease | 3310 | 4.0 |
| Late effects of Cereberovascular disease | 438 | 3.7 |
| History of fall | V1588 | 3.6 |
| Symptoms involving urinary system | 788 | 3.2 |
| Stress incontinence, female | 6256 | 3.2 |
| Urinary Tract infection | 5990 | 3.2 |
| Delirium due to conditions classified elsewhere | 2930 | 3.2 |
| Unspecified Fall | E8889 | 3.2 |
| Contusion of face, scalp, and neck except eye(s) | 920 | 3.2 |
| Contusion of eye and adnexa | 921 | 3.2 |
| Hematuria | 5997 | 3.0 |
| Bacterial infection in conditions classified elsewhere and of unspecified site | 41 | 2.9 |
| Other bacterial pneumonia | 482 | 2.9 |
| Pneumonia due to other specified organism | 483 | 2.9 |
| Altered mental status | 78097 | 2.7 |
| Abnormality of gait | 7812 | 2.6 |
| Other and ill-defined cerebrovascular disease | 437 | 2.6 |
| Convulsions | 7803 | 2.6 |
| Alterations of consciousness | 7800 | 2.5 |
| Other complications due to genitourinary device, implant, and graft | 99676 | 2.4 |
| Concussion | 850 | 2.4 |
| Cerebral laceration and contusion | 851 | 2.4 |
| Subarachnoid subdural and extradural hemorrhage following injury | 852 | 2.4 |
| Other and unspecified intracranial hemorrhage following injury | 853 | 2.4 |
| Intracranial injury of other and unspecified nature | 854 | 2.4 |
| Fracture of clavicle | 810 | 2.3 |
| Fracture of scapula | 811 | 2.3 |
| Fracture of humerus | 812 | 2.3 |
| Ill-defined fractures of upper limb | 818 | 2.3 |
| Disorders of fluid electrolyte and acid-base balance | 276 | 2.3 |
| Other and unspecified disorders of joint | 719 | 2.3 |
| Senility without mention of psychosis | 797 | 2.2 |
| Care involving use of rehabilitation procedures | V57 | 2.1 |
| Unspecified senile psychotic condition | 2909 | 2.1 |
| Unavailability of other medical facilities for care | V63 | 2.0 |
| Vascular Dementia | 2904 | 2.0 |
| Contusion of lower limb and of other and unspecified sites | 924 | 2.0 |
| Other cellulitis and abscess | 682 | 2.0 |
| Blindness and low vision | 369 | 1.9 |
| Deficiency of b-complex components | 266 | 1.9 |
| Other psychosocial circumstances | V62 | 1.8 |
| Parkinson's disease | 332 | 1.8 |
| Syncope and collapse | 7802 | 1.8 |
| Fracture of rib(s) sternum larynx and trachea | 807 | 1.8 |
| Functional digestive disorders not elsewhere classified | 564 | 1.8 |
| Acute renal failure | 584 | 1.8 |
| Pressure ulcer | 7070 | 1.7 |
| Pressure ulcer stages | 7072 | 1.7 |
| Carrier of infectious disease | V02 | 1.7 |
| Ulcer of lower limbs, except decubitus ulcer | 7071 | 1.6 |
| Hallucinations | 7801 | 1.6 |
| Duodenal ulcer | 532 | 1.6 |
| Hypotension | 458 | 1.6 |
| Unspecified renal failure | 586 | 1.6 |
| Sepsis | 99591 | 1.6 |
| Septicemia | 38 | 1.6 |
| Personal history of certain other diseases | V12 | 1.5 |
| Other diseases of lung | 5188 | 1.5 |
| Osteoarthrosis and allied disorders | 715 | 1.5 |
| Epilepsy and recurrent seizures | 345 | 1.5 |
| Osteoporosis | 7330 | 1.4 |
| Fracture of other parts of femur | 821 | 1.4 |
| Fracture of neck of femur | 820 | 1.4 |
| Fracture of Pelvis | 808 | 1.4 |
| Fracture of vertebral column without mention of spinal cord injury | 805 | 1.4 |
| Other disorders of pancreatic internal secretion | 251 | 1.4 |
| Abnormal results of function studies | 794 | 1.4 |
| Chronic kidney disease | 585 | 1.4 |
| Retention of urine | 7882 | 1.3 |
| Other unknown and unspecified cause of morbidity and mortali | 7999 | 1.3 |
| Other disorders of kidney and ureter | 593 | 1.3 |
| Urinary incontinence, unspecified | 78830 | 1.2 |
| Other cerebral degenerations (excluding Alzheimer’s) | 3311 | 1.2 |
| Other cerebral degenerations (excluding Alzheimer’s) | 3312 | 1.2 |
| Other cerebral degenerations (excluding Alzheimer’s) | 3313 | 1.2 |
| Other cerebral degenerations (excluding Alzheimer’s) | 3314 | 1.2 |
| Other cerebral degenerations (excluding Alzheimer’s) | 3315 | 1.2 |
| Other cerebral degenerations (excluding Alzheimer’s) | 3316 | 1.2 |
| Other cerebral degenerations (excluding Alzheimer’s) | 3317 | 1.2 |
| Other cerebral degenerations (excluding Alzheimer’s) | 3318 | 1.2 |
| Other cerebral degenerations (excluding Alzheimer’s) | 3319 | 1.2 |
| Other and unspecified injury to head face and neck | 9590 | 1.2 |
| Nervousness | 7992 | 1.2 |
| Transient cerebral ischemia | 435 | 1.2 |
| Other specified housing or economic circumstances | V6089 | 1.1 |
| Other disorders of soft tissues | 729 | 1.1 |
| Accidental fall from bed | E8844 | 1.1 |
| Other open wound of head | 873 | 1.1 |
| Intestinal infections due to other organisms | 8 | 1.1 |
| Infectious colitis, enteritis, and gastroenteritis | 9 | 1.1 |
| Pneumonia, organism unspecified | 486 | 1.1 |
| Bronchopneumonia, organism unspecified | 485 | 1.1 |
| Pneumococcal pneumonia | 481 | 1.1 |
| Pneumonitis due to solids and liquids | 507 | 1.0 |
| Other speech disturbance | 7845 | 1.0 |
| Vitamin D deficiency | 268 | 1.0 |
| Artificial opening status | V44 | 1.0 |
| Gangrene | 7854 | 1.0 |
| Symptoms concerning nutrition metabolism and development | 783 | 0.9 |
| Other specified forms of hearing loss | 3898 | 0.9 |
| Accidental fall on or from stairs or steps | E880 | 0.9 |
| Fall on same level from slipping, tripping and stumbling | E885 | 0.9 |
| Thyrotoxicosis with or without goiter | 242 | 0.9 |
| Kyphoscoliosis and scoliosis | 7373 | 0.9 |
| Dysphagia | 7872 | 0.8 |
| Dependence on other enabling machines | V468 | 0.8 |
| Infection with microorganisms resistant to other specified antimycobacterial agents | V097 | 0.8 |
| Pathologic fracture | 7331 | 0.8 |
| Gastrointestinal hemorrhage | 578 | 0.8 |
| Gastrointestinal mucositis (ulcerative) | 538 | 0.8 |
| Other specified disorders of intestine | 56989 | 0.8 |
| Unspecified disorder of intestine | 5699 | 0.8 |
| Cerebral thrombosis with cerebral infarction | 43401 | 0.8 |
| Cerebral embolism with cerebral infarction | 43411 | 0.8 |
| Cerebral artery occlusion, unspecified with cerebral infarction | 43491 | 0.8 |
| Calculus of kidney and ureter | 592 | 0.7 |
| Alcohol dependence syndrome | 303 | 0.7 |
| Nondependent alcohol abuse | 3050 | 0.7 |
| Other procedures without mention of misadventure at the time of procedure as the cause of abnormal reaction of patient or of later complication | 879 | 0.7 |
| Tachycardia, unspecified | 7850 | 0.7 |
| Palpitations | 7851 | 0.7 |
| Other abnormal heart sounds | 7853 | 0.7 |
| Other diseases of respiratory system, not elsewhere classified | 5198 | 0.7 |
| Problems related to lifestyle | V69 | 0.6 |
| Other abnormal findings of blood chemistry | 7906 | 0.6 |
| Other personal history presenting hazards to health | V15 | 0.5 |
| Open wound of elbow forearm and wrist | 881 | 0.5 |
| Major depressive disorder single episode | 2962 | 0.5 |
| Major depressive disorder recurrent episode | 2963 | 0.5 |
| Spinal stenosis other than cervical | 7240 | 0.5 |
| Spinal stenosis in cervical region | 7230 | 0.5 |
| Disorders of mineral metabolism | 275 | 0.4 |
| Other specified local infections of skin and subcutaneous tissue (approximate match) | 6868 | 0.4 |
| Other and unspecified anemias | 285 | 0.4 |
| Other local infections of skin and subcutaneous tissue | 686 | 0.4 |
| Nausea and vomiting | 7870 | 0.3 |
| Other and unspecified noninfectious gastroenteritis and colitis | 558 | 0.3 |
| Fever and other physiologic disturbances of temperature regulation | 7806 | 0.1 |

ICD, International Classification of Diseases; CM, Clinical Classification.

Supplementary Table 6. ICD-10 codes used to assess the Hospital Frailty Risk Score (HFRS).

| ICD-10 Condition | ICD-10-CM | Points |
| --- | --- | --- |
| Dementia in Alzheimer's disease | F00 | 7.1 |
| Hemiplegia | G81 | 4.4 |
| Alzheimer's disease | G30 | 4.0 |
| Sequelae of cerebrovascular disease (secondary codes) | I69 | 3.7 |
| Other symptoms and signs involving the nervous and musculoskeletal systems (R29·6 Tendency to fall) | R29 | 3.6 |
| Other disorders of urinary system (includes urinary tract infection and urinary incontinence) | N39 | 3.2 |
| Delirium, not induced by alcohol and other psychoactive substances | F05 | 3.2 |
| Unspecified fall | W19 | 3.2 |
| Superficial injury of head | S00 | 3.2 |
| Unspecified haematuria | R31 | 3.0 |
| Other bacterial agents as the cause of diseases classified to other chapters (secondary code) | B96 | 2.9 |
| Other symptoms and signs involving cognitive functions and awareness | R41 | 2.7 |
| Abnormalities of gait and mobility | R26 | 2.6 |
| Other cerebrovascular diseases | I67 | 2.6 |
| Convulsions, not elsewhere classified | R56 | 2.6 |
| Somnolence, stupor and coma | R40 | 2.5 |
| Complications of genitourinary prosthetic devices, implants and grafts | T83 | 2.4 |
| Intracranial injury | S06 | 2.4 |
| Fracture of shoulder and upper arm | S42 | 2.3 |
| Other disorders of fluid, electrolyte and acid- base balance | E87 | 2.3 |
| Other joint disorders, not elsewhere classified | M25 | 2.3 |
| Volume depletion | E86 | 2.3 |
| Senility | R54 | 2.2 |
| Care involving use of rehabilitation procedures | Z50 | 2.1 |
| Unspecified dementia | F03 | 2.1 |
| Other fall on same level | W18 | 2.1 |
| Problems related to medical facilities and other health care | Z75 | 2.0 |
| Vascular dementia | F01 | 2.0 |
| Superficial injury of lower leg | S80 | 2.0 |
| Cellulitis | L03 | 2.0 |
| Blindness and low vision | H54 | 1.9 |
| Deficiency of other B group vitamins | E53 | 1.9 |
| Problems related to social environment | Z60 | 1.8 |
| Parkinson's disease | G20 | 1.8 |
| Syncope and collapse | R55 | 1.8 |
| Fracture of rib(s), sternum and thoracic spine | S22 | 1.8 |
| Other functional intestinal disorders | K59 | 1.8 |
| Acute renal failure | N17 | 1.8 |
| Decubitus ulcer | L89 | 1.7 |
| Carrier of infectious disease | Z22 | 1.7 |
| Streptococcus and staphylococcus as the cause of diseases classified to other chapters | B95 | 1.7 |
| Ulcer of lower limb, not elsewhere classified | L97 | 1.6 |
| Other symptoms and signs involving general sensations and perceptions | R44 | 1.6 |
| Duodenal ulcer | K26 | 1.6 |
| Hypotension | I95 | 1.6 |
| Unspecified renal failure | N19 | 1.6 |
| Other septicaemia | A41 | 1.6 |
| Personal history of other diseases and conditions | Z87 | 1.5 |
| Respiratory failure, not elsewhere classified | J96 | 1.5 |
| Exposure to unspecified factor | X59 | 1.5 |
| Other arthrosis | M19 | 1.5 |
| Epilepsy | G40 | 1.5 |
| Osteoporosis without pathological fracture | M81 | 1.4 |
| Fracture of femur | S72 | 1.4 |
| Fracture of lumbar spine and pelvis | S32 | 1.4 |
| Other disorders of pancreatic internal secretion | E16 | 1.4 |
| Abnormal results of function studies | R94 | 1.4 |
| Chronic renal failure | N18 | 1.4 |
| Retention of urine | R33 | 1.3 |
| Unknown and unspecified causes of morbidity | R69 | 1.3 |
| Other disorders of kidney and ureter, not elsewhere classified | N28 | 1.3 |
| Unspecified urinary incontinence | R32 | 1.2 |
| Other degenerative diseases of nervous system, not elsewhere classified | G31 | 1.2 |
| Nosocomial condition | Y95 | 1.2 |
| Other and unspecified injuries of head | S09 | 1.2 |
| Symptoms and signs involving emotional state | R45 | 1.2 |
| Transient cerebral ischaemic attacks and related syndromes | G45 | 1.2 |
| Problems related to care-provider dependency | Z74 | 1.1 |
| Other soft tissue disorders, not elsewhere classified | M79 | 1.1 |
| Fall involving bed | W06 | 1.1 |
| Open wound of head | S01 | 1.1 |
| Other bacterial intestinal infections | A04 | 1.1 |
| Diarrhoea and gastroenteritis of presumed infectious origin | A09 | 1.1 |
| Pneumonia, organism unspecified | J18 | 1.1 |
| Pneumonitis due to solids and liquids | J69 | 1.0 |
| Speech disturbances, not elsewhere classified | R47 | 1.0 |
| Vitamin D deficiency | E55 | 1.0 |
| Artificial opening status | Z93 | 1.0 |
| Gangrene, not elsewhere classified | R02 | 1.0 |
| Symptoms and signs concerning food and fluid intake | R63 | 0.9 |
| Other hearing loss | H91 | 0.9 |
| Fall on and from stairs and steps | W10 | 0.9 |
| Fall on same level from slipping, tripping and stumbling | W01 | 0.9 |
| Thyrotoxicosis [hyperthyroidism] | E05 | 0.9 |
| Scoliosis | M41 | 0.9 |
| Dysphagia | R13 | 0.8 |
| Dependence on enabling machines and devices | Z99 | 0.8 |
| Agent resistant to penicillin and related antibiotics | U80 | 0.8 |
| Osteoporosis with pathological fracture | M80 | 0.8 |
| Other diseases of digestive system | K92 | 0.8 |
| Cerebral Infarction | I63 | 0.8 |
| Calculus of kidney and ureter | N20 | 0.7 |
| Mental and behavioural disorders due to use of alcohol | F10 | 0.7 |
| Other medical procedures as the cause of abnormal reaction of the patient | Y84 | 0.7 |
| Abnormalities of heart beat | R00 | 0.7 |
| Unspecified acute lower respiratory infection | J22 | 0.7 |
| Problems related to life-management difficulty | Z73 | 0.6 |
| Other abnormal findings of blood chemistry | R79 | 0.6 |
| Personal history of risk-factors, not elsewhere classified | Z91 | 0.5 |
| Open wound of forearm | S51 | 0.5 |
| Depressive episode | F32 | 0.5 |
| Spinal stenosis (secondary code only) | M48 | 0.5 |
| Disorders of mineral metabolism | E83 | 0.4 |
| Polyarthrosis | M15 | 0.4 |
| Other anaemias | D64 | 0.4 |
| Other local infections of skin and subcutaneous tissue | L08 | 0.4 |
| Nausea and vomiting | R11 | 0.3 |
| Other noninfective gastroenteritis and colitis | K52 | 0.3 |
| Fever of unknown origin | R50 | 0.1 |

ICD, International Classification of Diseases; CM, Clinical Classification.

Supplementary Table 7. The five most frequent principal diagnoses of hospitalizations in patients with metastatic and non-metastatic CRC (before PSM).

|  | **Metastatic disease** | | **Non-metastatic disease** | |
| --- | --- | --- | --- | --- |
| Rank | **ICD-9-CM Codes** | **Description** | **ICD-9-CM Codes** | **Description** |
| 1 | 197.7 | Secondary malignant neoplasm of liver | 153.6 | Malignant neoplasm of ascending colon |
| 2 | 153.9 | Malignant neoplasm of colon, unspecified site | 154.1 | Malignant neoplasm of rectum |
| 3 | 153.3 | Malignant neoplasm of sigmoid colon | 153.4 | Malignant neoplasm of cecum |
| 4 | 153.4 | Malignant neoplasm of cecum | 153.3 | Malignant neoplasm of sigmoid colon |
| 5 | V58.11 | Encounter for antineoplastic chemotherapy | 486 | Pneumonia, organism unspecified |

PSM, propensity-score matching; ICD, International Classification of Disease; CM, Clinical Modification; CRC, colorectal cancer.
